# High Magnetic Field Stability in a Planar Graphene-NbSe_2_ SQUID

**DOI:** 10.1021/acs.nanolett.3c01552

**Published:** 2023-06-22

**Authors:** Ayelet Zalic, Takashi Taniguchi, Kenji Watanabe, Snir Gazit, Hadar Steinberg

**Affiliations:** † The Racah Institute of Physics, The Hebrew University of Jerusalem, Jerusalem 91904, Israel; ‡ The Center for Nanoscience and Nanotechnology, Hebrew University of Jerusalem, Jerusalem 91904, Israel; § International Center for Materials Nanoarchitectonics, National Institute for Materials Science, 1-1 Namiki, Tsukuba 305-0044, Japan; ∥ Research Center for Functional Materials, National Institute for Materials Science, 1-1 Namiki, Tsukuba 305-0044, Japan; ⊥ The Fritz Haber Research Center for Molecular Dynamics, The Hebrew University of Jerusalem, Jerusalem 91904, Israel

**Keywords:** graphene, NbSe_2_, Josephson interference, planar SQUID, high magnetic field

## Abstract

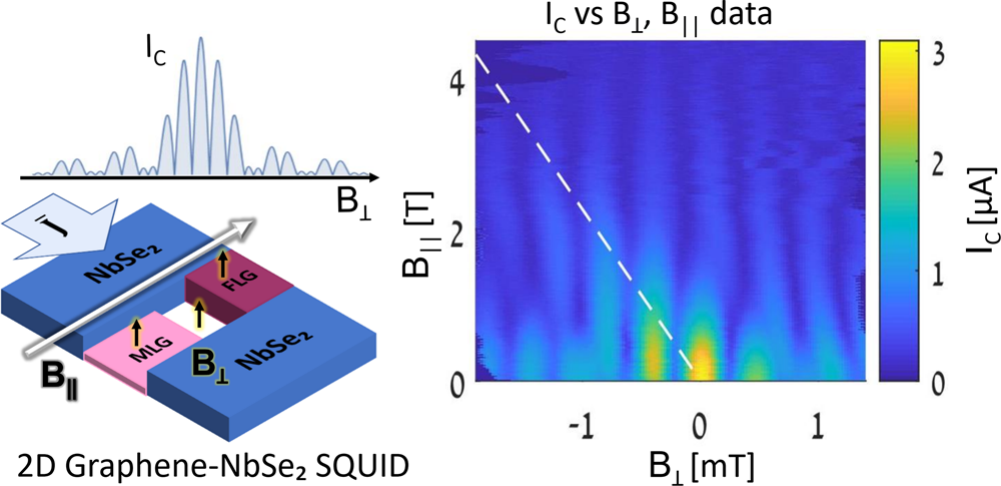

Thin NbSe_2_ retains superconductivity at a high in-plane magnetic field up to 30 T. In this work we construct a novel atomically thin, all van der Waals SQUID, in which current flows between NbSe_2_ contacts through two parallel graphene weak links. The 2D planar SQUID remains uniquely stable at high in-plane field, which enables tracing critical current interference patterns as a function of the field up to 4.5 T. From these we extract the evolution of the current distribution up to high fields, demonstrating sub-nanometer sensitivity to deviation of current flow from a perfect atomic plane and observing a field-driven transition in which supercurrent redistributes to a narrow channel. We further suggest a new application of the asymmetric SQUID geometry to directly probe the current density in the absence of phase information.

Transition-metal dichalcogenide (TMD) superconductors such as NbSe_2_ can be mechanically exfoliated to yield thin layers down to the monolayer limit.^1,2^ Thin NbSe_2_ superconducting electrodes sustain very high in-plane magnetic fields (*B*_∥_) beyond the Pauli limit, due to suppressed orbital depairing and Ising spin–orbit coupling (ISOC), which locks spins in the out-of-plane orientation.^1^ The superconducting gap persists nearly unchanged up to 10 T^3^ and remains observable up to 25 T in tunneling measurements.^4^

It is useful to incorporate thin TMD superconductors in devices that utilize their unique properties at high *B*_∥_. NbSe_2_ has been coupled laterally to graphene to realize NS junctions.^5,6^ Devices consisting of NbSe_2_ flakes coupled on both sides of a narrow graphene channel (Figure 1a) are well-behaved Josephson junctions (JJs).^7,8^ Our two-dimensional planar Josephson junctions (2DJJs), constructed exclusively from van der Waals (vdW) materials by transferring a cracked NbSe_2_ flake on top of graphene, are unique in retaining a Josephson effect at high parallel magnetic fields.

**Figure 1:**
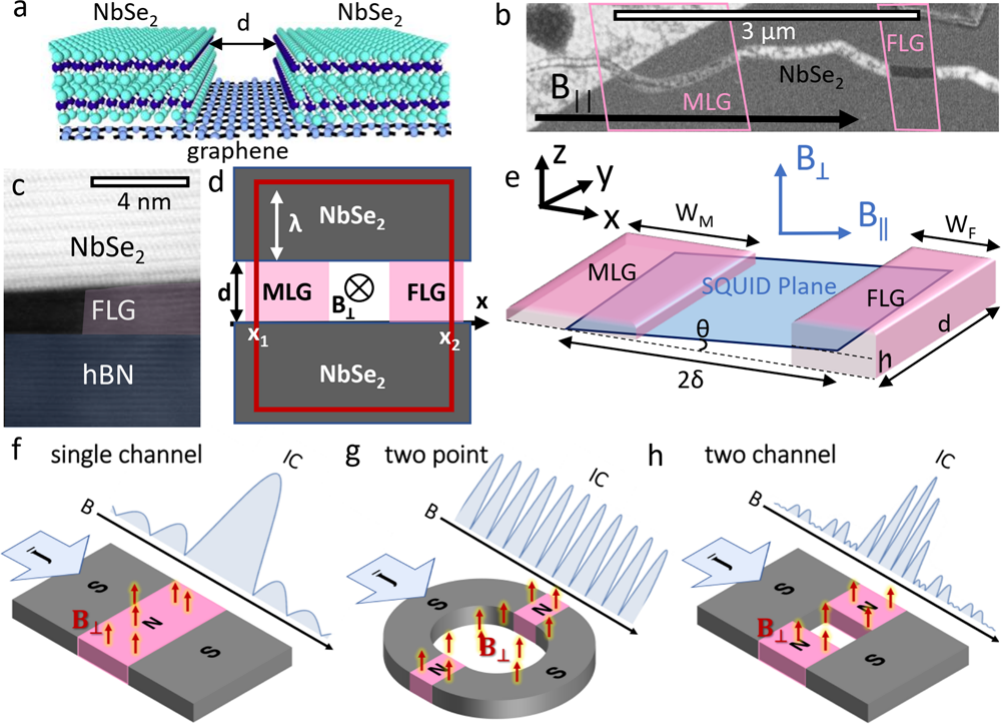
(a) Planar NbSe_2_-graphene-NbSe_2_ JJ geometry. (b) SEM image of the device. The conducting NbSe_2_ appears as a smooth dark gray region with a crack in the middle. An hBN flake used to pick up the cracked NbSe_2_ covers the top-left corner, while the hBN substrate is visible inside the crack. Both are insulators and appear white and grainy in SEM due to charging. Bridging the crack, the MLG flake appears light gray and the FLG region as dark gray. Pink outlines of MLG and FLG flakes extending beneath NbSe_2_ are overlaid from an optical microscope image prior to stacking. (c) False-color cross-sectional TEM measurement of the FLG region showing atomically clean interfaces between NbSe_2_ and FLG and between FLG and hBN. (d) Schematic showing *B*_⊥_ flux through one possible current circulation path, with an area (2λ + *d*)|*x*_2_ – *x*_1_|. (e) Schematic illustration of FLG and MLG parallel weak links of different thicknesses. Directions *x̂* and *ŷ* are in the plane of the flakes, and *ẑ* is perpendicular. The mean SQUID plane is shown in blue, at an angle θ*. B*_∥_ is parallel to the SQUID plane, and *B*_⊥_ is perpendicular to it. Crack length *d* is in the direction of current flow (f–h) Illustration of interference patterns for different junction geometries: (f) single channel (Fraunhofer), (g) two point (SQUID), and (h) two channel (Fraunhofer envelope modulates SQUID oscillation).

In this work, we extend the all-vdW 2DJJ concept to a SQUID geometry, with current flowing between NbSe_2_ contacts through parallel monolayer graphene (MLG) and few-layer graphene (FLG) weak links (see Figure 1b). In this structure, the graphene flakes are supported by a flat, insulating hexagonal boron nitride (hBN) substrate, and all interfaces are atomically clean (Figure 1c), ensuring a planar geometry. The stability of the SQUID in magnetic field allows us to trace the evolution of *I*_C_(*B*_⊥_) interference patterns up to *B*_∥_ = 4.5 T. We reconstruct the distribution of current flow from *I*_C_(*B*_⊥_), presenting a new method utilizing the asymmetric SQUID geometry without the need for phase retrieval. At high *B*_∥_, we observe a qualitatively apparent transition, indicating a narrowing of the current channel in the MLG. Furthermore, we find that our SQUID is highly sensitive to the nanometer-scale height difference between the MLG and FLG current planes.

For our SQUID we use a single cracked NbSe_2_ flake, of approximately 13 nm thickness, as seen in the cross-sectional TEM measurement (see the Supporting Information); the length of both junctions, imposed by the NbSe_2_ crack, is *d* = 140 nm in the direction of the current flow (see Figure 1d). It is important to distinguish between different planes of reference in the sample. The “in-plane” magnetic field *B*_∥_ is defined as oriented parallel to the mean SQUID plane: the plane connecting the centers of the MLG and FLG flakes (Figure 1e). This plane is at a small angle, θ, with respect to the plane of the MLG flake.

We begin by showcasing the basic properties of 2D SQUID in Figure 2. Current–voltage characteristics of the SQUID switch from zero to finite resistance at the critical current *I*_C_, which we define according to a voltage threshold (Figure 2a). The transition from superconducting to normal conductance is sharpest at *B*_∥_ = 0 T. Figure 2b illustrates the modulation of the critical current by varying the charge carrier density. In our SQUID the common back-gate tunes both FLG and MLG densities simultaneously; therefore, it is not possible to pinpoint the MLG Dirac point exactly (it is likely in the region of minimal *I*_C_ around 10 V).

**Figure 2:**
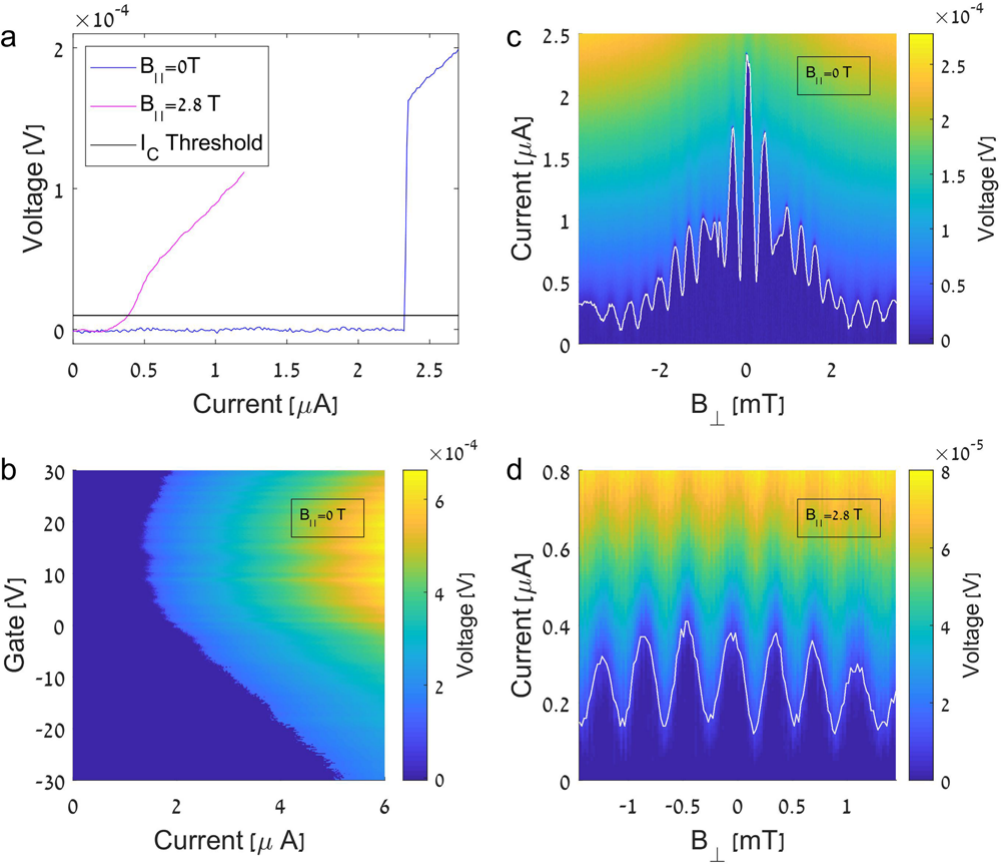
(a) Current–voltage traces at *B*_∥_ = 0 T, *B*_∥_ = 2.8 T, and zero gate voltage. The voltage threshold for determining critical current is shown in black. (b) Current–voltage traces as a function of gate voltage at zero field. Gate modulates the critical current, with a minimum at around 10 V (the MLG Dirac point) (c) Interference pattern at *B*_∥_ = 0 T, with a Δ*B* ≈ 380 μT oscillation corresponding to the SQUID area and an envelope reflecting the area of the MLG and FLG junctions. A white line marks the threshold detection of *I*_C_. (d) Interference pattern at *B*_∥_ = 2.8 T. The SQUID oscillation maintains periodicity similar to that of (a), but the envelope is no longer visible. Note that the measured *B*_⊥_ range at *B*_∥_ = 2.8 T is smaller than at 0 T to avoid entry of vortices.

Upon application of a small (mT scale for our devices) magnetic field perpendicular to the junction (*B*_⊥_), the superconducting order parameter Δ*e*^*i*φ^ acquires a position-dependent phase and undergoes interference. This leads to a Fourier relation between the critical current *I*_C_(*B*_⊥_) and the maximal local critical current density *J*_0_(*x*)^9^
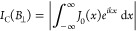
1where , such that a loop connecting any two points *x*_1_, *x*_2_ and extending across the junction length *d* into the superconductors up to the London penetration depth λ encloses a magnetic flux of *k*(*x*_2_– *x*_1_)/2π in units of Φ_0_ (see Figure 1d).

The interference pattern of *I*_C_(*B*_⊥_) at zero gate voltage and *B*_∥_ = 0 T is shown in Figure 2c. The rapid oscillations of *I*_C_, with a magnetic field period Δ*B* = Φ_0_*A*_SQ_ ≈ 380 μT, reflect the area of the SQUID *A*_SQ_ = 2δ(2λ + *d*) = 5.4 μm^2^. This area implies an effective penetration length λ = 930 nm, longer than λ_L_ ≈ 200 nm yet shorter than the Pearl length Λ = 2λ_L_^2^/*t* ≈ 6 μm. The SQUID oscillations are modulated by an envelope which derives from the areas of the MLG and FLG channels, as illustrated by a schematic of two channel interference in Figure 1h. Note that the measured *B*_∥_ = 0 T pattern is not perfectly symmetrical with respect to *B*_⊥_. This could be a signature of various symmetry-breaking effects^15,16^ but is most likely due to vortices in the vicinity of the junction or a small trapped parallel flux.

The introduction of *B*_∥_ dramatically changes this pattern. At *B*_∥_ = 2.8 T (Figure 2d), the SQUID oscillation persists and maintains its periodicity, whereas the envelope is no longer discernible. The data now resemble the two-point interference pattern in Figure 1g. The contrast between zero- and high-field interference patterns is one of the main results of our work. The transition to a two-point SQUID indicates a change in the supercurrent distribution, which becomes focused within a narrow channel at higher fields.

To gain initial insight into the expected *I*_C_(*B*_⊥_) in the SQUID, we make two simplifying assumptions: (i) the phase dynamics are local and (ii) the current–phase relation is sinusoidal. Both assumptions are typical for graphene-based JJs. However, ballistic graphene JJs may exhibit measurable skewness in the current–phase relation,^12^ while in ultrathin superconducting contacts, the Pearl length Λ = 2λ_L_^2^/*t* replaces λ_L_ as the relevant field decay scale and the dynamics become potentially nonlocal.^13^ Our smaller than bulk SQUID periodicity Δ*B*, discussed above, might hint at nonlocal dynamics; however, the typical nonlocal geometric relation Δ*B* = 1.8Φ_0_/*W*_S_^2^,^13^ with *W*_S_ being the width of the superconductor, is too large for our measured periodicity. This raises the need for future geometry-controlled experiments.

Using the assumptions described above, we begin by approximating a spatially uniform current density in each channel. Applying eq 1 to this simplified model produces a two-channel diffraction pattern^14^ (see Figure 1h), with the finite FLG/MLG widths (*W*_F_, *W*_M_) generating Fraunhofer-like envelopes (Figure 1f) modulating the SQUID oscillations (Figure 1g). We describe the exact expression and the calculation leading to it in the Supporting Information. Below, we refer to this as the “Analytical Model”.

To investigate the supercurrent distribution systematically, we first turn to study how the *B*_∥_ = 0 T interference pattern evolves with respect to the applied gate voltage. We measure *I*_C_ as a function of *B*_⊥_ and *V*_G_ continuously, as shown in the color plot in Figure 3a. The overall SQUID periodicity remains fairly constant, whereas the critical current magnitude and the envelope both evolve with the gate voltage—indicating a variation in current distribution. Figure 3d shows selected interference patterns from Figure 3a at *V*_G_ = −30, 0, 30 V.

**Figure 3:**
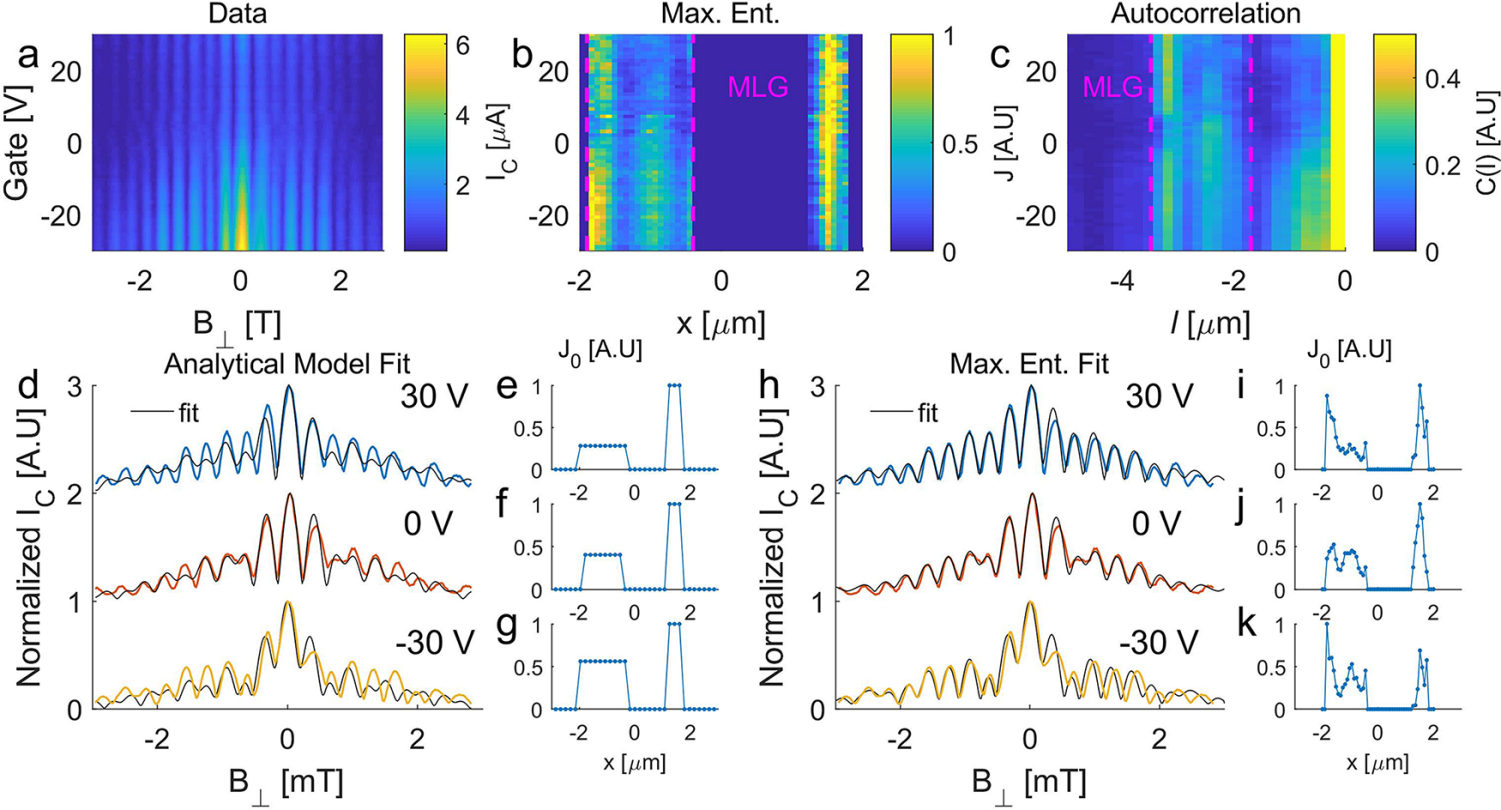
(a) *I*_C_ (color scale) vs *B*_⊥_ and gate voltage. (b) Normalized current density extracted from (a) using a maximum entropy method (see text). (c) Normalized autocorrelation function (see text) of the current density (color scale) vs *l*, the autocorrelation shift in the *x* coordinate. Compare the sideband bounded in pink dashed lines, which is proportional to the MLG current density convolved with the narrow FLG current channel, to the MLG current density bounded in pink lines in (b). (d) Interference patterns at gate voltages of −30, 0, and 30 V with two-channel analytical fit. (e–g) Current density profiles corresponding to the analytical fits in (d). (h) Interference patterns at gate voltages of −30, 0, and 30 V with maximum entropy fit. (i–k) Current density profiles corresponding to fits in (h).

We fit these curves using the analytical model described above, where the free parameters are the MLG and FLG widths *W*_M_, *W*_F_, the distance between their centers 2δ (see Figure 1e), and the ratio between their critical current densities *J*_M_/*J*_F_ (see the Supporting Information for details). Figure 3e–g shows the current density profile for each *V*_G_ trace extracted from the fit. For 2λ + *d* = 2 μm, we find that fit parameters *W*_M_, *W*_F_, δ agree with the dimensions determined from SEM measurements shown in Figure 1d (see comparison table in the Supporting Information). The extracted current densities in Figure 3e–g show that the MLG current modulates strongly with gate, increasing at negative gate voltage.

We now turn to a detailed investigation of the field-driven transition shown in Figure 2, tracing the interference patterns continuously with increasing *B*_∥_. In Figure 4a we plot *I*_C_(*B*_⊥_,*B*_∥_), at *V*_G_ = 0 V. This interference plot is extremely stable, up to *B*_∥_ = 4 T, barring minor flux jumps around *B*_∥_ = 2.8 T, likely due to vortices entering the vicinity of the junction (see the Supporting Information). *B*_∥_ is kept strictly aligned to the SQUID plane by careful compensation of the out-of-plane coil. This precise alignment procedure utilizes the phase of the fast SQUID oscillations and allows us to avoid flux jumps up to higher fields than in previous works.^8,27^*B*_⊥_ is defined as geometrically perpendicular to *B*_∥_. Note that we control the field along the axes of the lab magnets, which are not exactly aligned with the SQUID plane; we describe the compensation and alignment procedure in detail in the Supporting Information.

**Figure 4:**
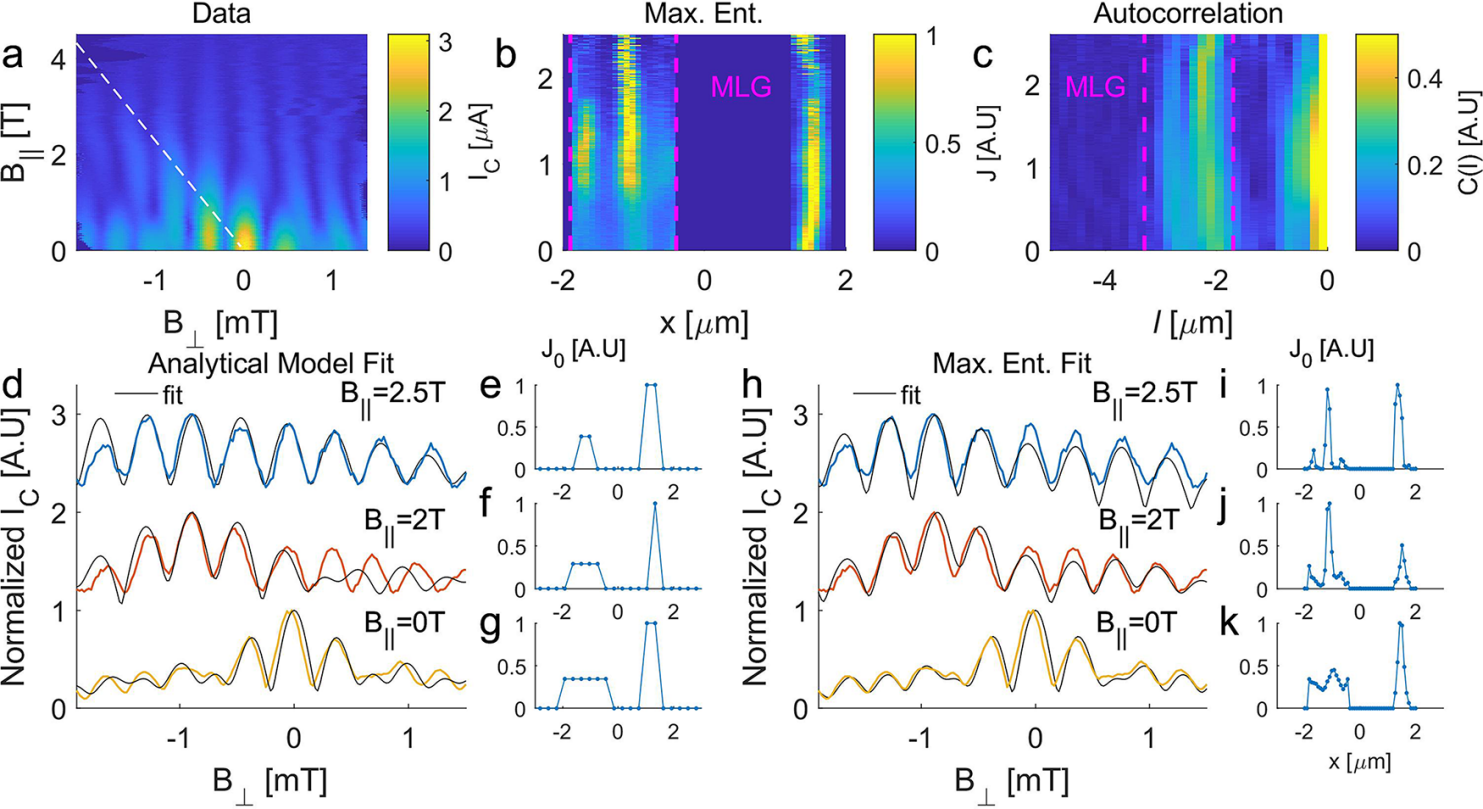
(a) *I*_C_ (color scale) vs *B*_⊥_ and *B*_∥_, at zero gate voltage. The white dashed line marks the geometric angle θ ≈ 0.025° between the SQUID plane and the MLG. (b) Normalized current density extracted using the maximum entropy method from the data in Figure S4. (c) Normalized autocorrelation function of the current density from (a). Compare the sideband to the MLG current density in (b). (d) Interference patterns at *B*_∥_ = 0, 2, and 2.5 T with two-channel analytical fit. (e–g) Current density profiles corresponding to the analytical fits in (d) (h) Interference patterns at *B*_∥_ = 0, 2, and 2.5 T with maximum entropy fit. (i–k) Current density profiles corresponding to fits in (h).

The data exhibit a diagonal drift of the MLG envelope toward negative *B*_⊥_, evident in a shift of the maximal *I*_C_ and of the first Fraunhofer nodes. This diagonal is due to deviation from a perfect planar geometry: the step height *h* between MLG and FLG planes creates an angle θ between the SQUID plane and the MLG. With *B*_∥_ aligned exactly to the SQUID plane, it contributes a component *B*_∥_(sinθ) of flux perpendicular to the MLG and FLG flakes. The condition of zero flux through the MLG, for which the Fraunhofer envelope function is maximal, thus drifts toward negative values of *B*_⊥_, following the linear relation *B*_⊥_(max(*I*_C_)) = −*B*_∥_(sinθ) to compensate (see full calculation the Supporting Information). The angle θ ≈ 0.025° extracted from the fit indicates a step height of around 1 nm, while the TEM measured FLG thickness is 2.4 nm; this probably indicates a distribution of current throughout the FLG, with the mean SQUID plane being determined by the center of the FLG flake. There could also be an additional step or curvature in the hBN outside the range of the TEM. The 2DJJ SQUID is thus an extremely sensitive tool for tracking deviations from the atomic planar geometry.

The effect of field-driven current redistribution is apparent in the transition to a SQUID-like interference pattern. Figure 4d shows a series of interference patterns at *B*_∥_ = 0, 2, and 2.5 T, together with the best fit of our analytical model. The current densities corresponding to the fit appear in Figure 4e–g, showing a narrower current profile in the MLG as *B*_∥_ increases.

The analytical curves fit the data reasonably well for the central lobes of the interference patterns (see Figures 3d and 4d), but the higher order lobes are far more pronounced in the data compared with the fit, hinting that the current density distribution has finer spatial detail beyond the two uniform conduction channels. We thus turn to extract the current distribution in greater detail. Since the interference pattern reflects the absolute value of the Fourier transform of the current density, phase information is lost and it is impossible to directly apply an inverse Fourier transform. The oft-cited Dynes–Fulton approach to phase retrieval assumes a nearly symmetric current distribution, and so is not applicable in our case.^17^

We use an approach that we term “the maximum entropy method” suitable for reproducing current distributions with no symmetry requirements. The method postulates a current density profile sampled at *N* discrete spatial points and subject to known physical constraints to calculate the critical current via a forward Fourier transform. The current density profile is then adjusted to obtain the best fit of the calculated interference pattern to the data, as in ref (18), with an additional maximum entropy constraint in order to avoid spurious sharp changes in current density.^19^ See the Supporting Information for the full details of our fitting algorithm, including our approach toward calibrating parameters and avoiding overfitting (based on the L curve^20^).

To demonstrate the maximum entropy method, we return to the interference patterns measured at *B*_∥_ = 0 T and different gate voltages (Figure 3), and extract the current densities at *V*_G_ = −30, 0 and 30 V, shown (normalized by the maximal *J*_0_ for each gate) in Figure 3i–k. To confirm self-consistency, we apply eq 1 to reproduce the interference pattern corresponding to the extracted current density. We compare these to the measured patterns in Figure 3h; the obtained fit is indeed far better than the analytical fit in Figure 3d, especially in the higher order lobes. In Figure 3i–k, we observe that the extracted current within the MLG is distributed with two peaks; this could be related to some device-specific feature, or perhaps these are the familiar graphene edge channels first observed in refs (10 and 11). The color plot in Figure 3b shows the full evolution of the extracted current density with the gate voltage.

The fit obtained by the maximum entropy method is remarkably successful. Nevertheless, it is a complex method with many algorithmic as well as physical parameters. Since we are interested in circumventing the phase-retrieval problem altogether, we introduce a new method that harnesses our asymmetric SQUID geometry. We employ the narrower FLG junction as a direct probe of the current density in the wider MLG junction. This method leans on the Wiener–Khinchin theorem, which states that the energy spectral density of a function and its autocorrelation *C*(*l*) are Fourier transform pairs. In our case, |*I*_C_(*B*_⊥_)|^2^ is the energy spectral density of *J*_0_(*x*) and thus
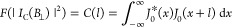
2

Note that here, to calculate the autocorrelation, we perform a forward Fourier transform of the energy spectral density, which does not require any knowledge of the phase. This calculation is always possible; however, only for a specific asymmetric SQUID geometry, the autocorrelation of *J*_0_(*x*) also provides direct information about *J*_0_(*x*) itself. Consider an ideal two-channel device, where the current density in the one channel is extremely narrow, approximated by the Dirac delta function, whereas the current in the other channel is widely distributed. The separation between the centers of the two channels is larger than their combined widths. The autocorrelation in this case contains a term equal to the current density in the wider channel (see calculations in the Supporting Information).

In our device, the FLG is a few times narrower than the MLG and carries a similar total current. In this instance, the autocorrelation convolves the FLG and MLG densities, resulting in a feature which qualitatively resembles the MLG current density “smeared” at the scale of the FLG width and centered at *l* = −2δ equal to the distance between the centers of the two channels. Figure 3c shows the autocorrelation as a function of gate; the resulting “sideband” centered at *l* = – 2.7 μm is qualitatively similar in its form to the extracted current density in Figure 3b. There is a clear qualitative agreement between the MLG current distributions extracted by maximum entropy and autocorrelation, shown between the pink lines in Figure 3b,c. This confirms the validity of these two very different methods.

We now apply these methods to extract a visual picture of the evolution of the current density with a parallel magnetic field. Figure 4b shows the current density extracted using the maximum entropy fit of an *I*_C_(*B*_⊥_,*B*_∥_) data set (see the Supporting Information). This map allows us to visualize how the current density in the MLG redirects into a narrow channel. The interference patterns produced by the maximum entropy procedure fit most of the measured patterns closely, as shown for selected values of *B*_∥_ = 0, 2, and 2.5 T in Figure 4h. The extracted current densities at this succession of fields are depicted in Figure 4i–k, illustrating again the narrowing of the current-carrying channel in the MLG as *B*_∥_ increases.

This phenomenology is apparent also in the current density extracted by autocorrelation. The sideband marked by pink lines in Figure 4c, centered around an autocorrelation shift *l* = 2.7 μm (equal to the distance between MLG and FLG channels), is qualitatively similar to the maximum entropy MLG current density shown in Figure 4b and also exhibits a narrowing of the current channel in the MLG commencing at *B*_∥_ = 2 T.

The transition toward narrow supercurrent channels has already been hinted at in our previous work—indeed, multiple diffusive MLG–NbSe_2_ junctions also undergo a transition to SQUID-like interference patterns where all lobes are of similar height at high *B*_∥_.^8^ In that work, the patterns were too disordered to fit to eq 1, and we could not rule out the role of ripples due to the SiO_2_ substrate.^8^ In the present work, the device is flat due to the use of an hBN substrate. In addition, the signal is sufficiently stable to allow a quantitative fitting. All models, assuming an experimental geometry corroborated by SEM and TEM, yield a clear transition between a distributed current density in the MLG at low fields, to a narrow supercurrent channel at high *B*_∥_.

We note that a similar effect of SQUID-like interference patterns at high *B*_∥_, seen by Suominen et al., was attributed to suppression of supercurrent in the bulk of the JJ due to a magnetic dipole formed by tilted flux lines.^21^ In our geometry, however, SQUID-like interference indicates one channel in the MLG, not necessarily on the edge. The flux focusing effect is also weaker in thin NbSe_2_ electrodes, where the tilt of the field lines is minimal due to a long London penetration length.

Thus in our devices, the origin of field-induced current redistribution is an open question. It suggests the existence of at least one conductance channel with resilience to high *B*_∥_. The suppression of a 2DJJ supercurrent vs *B*_∥_ is determined by the interplay of the Thouless and Zeeman energy scales,^8,22^ the Thouless energy being a transport energy scale determined by the inverse of the traversal time of the junction.^23^ Hence, the superior resilience of a single channel could be the consequence of a higher Thouless energy if a particular channel allows faster traversal of the junction. This could be, for example, a guided edge mode or a shorter channel in a nonuniform junction geometry. However, the presence of a similar effect in a number of devices in ref (8) suggests that it is not related to a particular geometry. Favored channels could also be the ones that experience minimal scattering in a disordered potential landscape. Alternatively, graphene could inherit Ising spin–orbit coupling by proximity to the NbSe_2_ within the extended contact region between the two materials.^24^ Such an interaction would enhance the stability of the carriers to the in-plane field, and spatial variation of the induced coupling could lead to preferred channels. All in all, we find that the in-plane magnetic field appears to create narrow superconducting channels in graphene-NbSe_2_ 2DJJs, an intriguing effect which has yet to be understood.

There has been a recent surge of interest in planar JJs with spin–orbit coupling,^25−28^ driven by predictions for topological effects tuned by parallel magnetic field.^29−32^ Looking to the future, further exploration of 2DJJs at high parallel field can shed light on the role of spin–orbit effects in the hybrid graphene-TMD structure.^24,33,34^

## Methods

We exfoliated hbN on markered SiO_2_ and located substrate flakes of thicknesses around 20–40 nm. We exfoliated graphene to SiO_2_ directly and NbSe_2_ first to PDMS and then stamped the PDMS on SiO_2_ to transfer the flakes. This method supplied large, thin flakes of NbSe_2_ that were not obtained by exfoliating directly from the blue tape to SiO_2_. We used an optical microscope to search for two long, narrow graphene flakes which are within a few μm distance of each other for the channels of the SQUID, as well as NbSe_2_ flakes that are a few layers thick and have an observable crack, less than 500 nm wide. We then employed a successive polycarbonate (PC) pickup technique^35^ to pick up first the NbSe_2_ and then the graphene strips oriented perpendicular to the crack and finally deposited the stack on the hBN substrate. We applied standard e-beam lithography and e-beam evaporation to create Ti/Au contacts to the NbSe_2_, removing surface oxide using in situ argon ion milling prior to evaporation. Four-probe measurements were conducted in a BluFors dilution cryostat with a 3 T/9 T vector magnet and a base temperature of 20 mK.

## Supporting Information Available

This work contains a Supporting Information document with the following chapters: The Supporting Information is available free of charge at https://pubs.acs.org/doi/10.1021/acs.nanolett.3c01552.

Parallel field alignment procedure, vortex penetration and device stability to out-of-plane flux, analytical calculation of two-channel interference pattern, maximum entropy reconstruction of the current profile via Markov Chain Monte Carlo simulated annealing, and current density extraction using the Wiener–Khinchin theorem (PDF)
