# Supplementary material for: High Magnetic Field Stability in a Planar Graphene-NbSe2 SQUID
Source: Nano Lett. 2023 Jun 22;23(13):6102–8. doi: 10.1021/acs.nanolett.3c01552 (PMC10347695; doi:10.1021/acs.nanolett.3c01552)
Supplement: Supplementary file 1 — nl3c01552_si_001.pdf [file nl3c01552_si_001.pdf]

High magnetic field stability in a planar graphene-$NbSe_2$ SQUID Supplemental Materials

Ayelet Zalic[1,2], Takashi Taniguchi[3], Kenji Watanabe[4], Snir Gazit[1,5], and Hadar Steinberg*[1,2]

[1]The Racah Institute of Physics, The Hebrew University of Jerusalem, Jerusalem 91904, Israel

[2]The Center for Nanoscience and Nanotechnology, Hebrew University of Jerusalem, Jerusalem 91904, Israel

[3]International Center for Materials Nanoarchitectonics, National Institute for Materials Science, 1-1 Namiki, Tsukuba 305-0044, Japan

[4]Research Center for Functional Materials, National Institute for Materials Science, 1-1 Namiki, Tsukuba 305-0044, Japan

[5]The Fritz Haber Research Center for Molecular Dynamics, The Hebrew University of Jerusalem, Jerusalem 91904, Israel

*Corresponding Author: Hadar Steinberg, email: hadar@phys.huji.ac.il

June 17, 2023

S1 Supplementary Section: Parallel field alignment procedure

Our SQUID is highly sensitive to perpendicular fields on the scale of tens of μT. This fact defines three relevant axes systems (see Fig. **S1**): the lab axis, along which we control B_z^{lab} and B_x^{lab} via our magnets; the SQUID axis, with $B_{||}$ parallel to the SQUID plane and $B_\perp$ perpendicular to it, and finally B_x, B_z which are parallel and perpendicular to the plane of the MLG, respectively. When applying magnetic field B_x^{lab} in the lab frame, stray perpendicular flux can penetrate the SQUID, either due to a small misalignment between the sample plane and the magnet axis, or from vortices or trapped magnetic flux in the leads, junction or the magnet itself. This leads to instability in the interference patterns, faster decay in the critical current with B_x^{lab}, and difficulty in interpretation of the results. We thus wish to apply high $B_{||}$ precisely along the SQUID plane, using the alignment procedure described below.

We calibrate $B = 0T$ in all directions and axes systems as zero applied field at cooldown. We then apply small B_z^{lab} and measure the interference pattern as a reference: at B_x^{lab}=0T, the interference

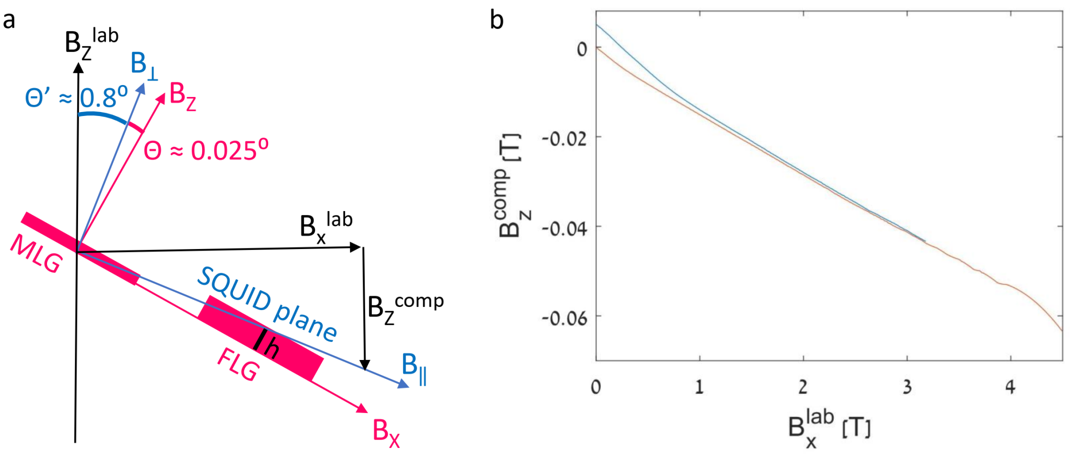

Supplementary Figure S1: **a.** Three axes systems: in black, the "lab" axes along which magnetic field is controlled. In blue, the SQUID plane, oriented at $\theta' = 0.8°$ with respect to the lab frame. In pink, the MLG plane, oriented at $\theta = 0.025°$ with respect to the SQUID plane. Applying B_x^{lab} together with the compensation field B_z^{comp} creates $B_{\parallel}$ in the SQUID plane **b.** B_z^{comp} as a function of B_x^{lab} for the data presented in Fig. **S3**c (blue) and main text Fig. 4 (orange)

pattern of the supercurrent has a clear maximum at B_z^{lab}=0T. Upon applying a small B_x^{lab} (say 10 mT) and subsequently measuring the interference pattern generated by B_z^{lab}, there will be a shift in the center of the pattern: since the center of the pattern is located at $B_\perp = 0T$, which no longer coincides with $B_z^{lab} = 0T$. To find the true state of zero flux through the SQUID, we assume a smooth evolution of the interference pattern with B_x^{lab}; that is, small changes in B_x^{lab} will cause a minimal change/shift in the interference pattern of the junction. Based on this assumption, we find the shift in B_z^{lab} which maximizes the cross-correlation (implemented using Matlab xcorr function) between the measurement at $B_x^{lab} = 10mT$ and the reference at B_x^{lab}=0T. This shift is the compensation field B_z^{comp}, which we then take to be true $B_\perp$=0T. The pattern at 10 mT then serves as a reference for calculating the compensation field at 20 mT and so on. Fig.**S1**b shows the resulting compensation field as a function of B_x^{lab} for the measurements in Fig. **S4**c and Fig. 4 of the main text. The compensation algorithm along with the entire measurement is automated.

The angle $\theta' \approx 0.8°$ between the z magnet axis and the normal to the SQUID plane is calculated from the ratio of the compensation field to B_x^{lab}. The angle between the MLG plane and the SQUID plane $\theta = 0.025°$ is then inferred from a fit to the interference pattern as described in Supplementary Section 2. In the main text, as well as what follows in the supplementary, we approximate $B_\parallel = \sqrt{(B_z^{comp})^2 + (B_x^{lab})^2}$, and $B_\perp = B_z^{lab} - B_z^{comp}$. These approximations are accurate at least until second order in the small parameters $\theta, \theta', \frac{B_\perp}{B_\parallel}$.

The downside of this complicated alignment procedure is that it can also compensate for shifts in the interference pattern due to relevant physical causes, such as changes in the ground phase of the junction leading to a shift in the phase of the interference pattern (a 0-π transition for example). This information will not be apparent in a plot of I_C vs $B_\perp, B_\parallel$, such as the one presented in Fig. 4 of the main text. However the information is not lost, since we track the applied compensation field as a function of B_x^{lab}

(see Fig. **S1**b). Note that there was trapped flux in the system in one of the measurements at $B_x^{lab} = 0T$, as evidenced by the finite compensation field. Note also that as B_x^{lab} approaches 4T, the compensation curve becomes non-linear; this is due to the automated cross-correlation algorithm gradually failing as the critical current descends below the minimal current detectable by the set voltage threshold.

S2 Supplementary Section: Vortex penetration and device stability to out-of-plane flux

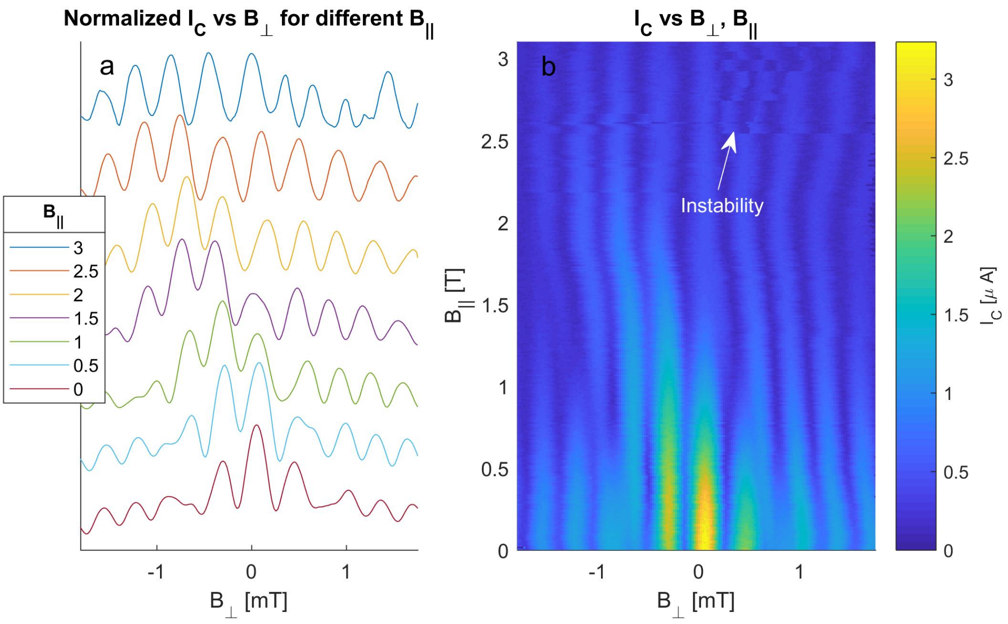

Supplementary Figure S2: **a.** An additional dataset showing critical current vs. $B_\perp$ at different values of $B_\parallel$ taken from the full map in panel (b.). The traces exhibit a diagonal drift of the Fraunhofer envelope as well as a transition to a SQUID-like lobe structure. The pattern remains stable up to 2.5 T; at 3T, it is disturbed, most likely by the entry of vortices **b.** The full map of $I_C(B_\perp, B_\parallel)$, showing the onset of instability above $B \parallel = 2.5T$

We present here another measurement nearly identical to the one shown in the main text in Fig. 4a, but with a wider range of $B_\perp$, thus containing a few more lobes in the interference pattern. We used this data set to extract the current density in main text Fig. 4b using the maximum entropy method (as shown in supplementary section 3), since the additional lobes improve the spatial resolution of the extracted current density. However, this measurement loses stability at $B_\parallel = 2.5T$, compared with the measurement shown in main text Fig. 4a which remains stable up to $B_\parallel = 4.5T$. Indeed, we performed around 10 similar measurement maps with different $B_\perp$ ranges, and found that in general, the narrower the range of $B_\perp$, the higher the values of $B_\parallel$ we could reach without incurring flux jumps. We attribute the flux jumps as arising from the penetration of vortices to the vicinity of the junction.

S3 Supplementary Section: Analytical calculation of two channel interference pattern

The Josephson effect occurs when current flows between two superconducting electrodes (in this case $NbSe_2$) connected by a weak link. The proximity of the superconductors, under the correct conditions, allows a supercurrent to flow through the junction. Upon application of magnetic field perpendicular to the junction, the superconducting order parameter $\Delta e^{i\varphi}$ acquires a position-dependent phase and undergoes interference, resulting in a diffraction pattern of the critical current in magnetic field. The first Josephson relation relates the critical current density $J(x)$ to the phase difference between the order parameters of the two superconductors A and B, $\gamma(x) = \varphi_B(x) - \varphi_A(x)$:

$$J(x) = J_0(x) sin(\gamma(x)) \tag{S1}$$

Here J_0 is the maximal possible critical current density at location x. The order parameter must retain a single-valued phase around any closed loop through which current may circulate, leading to the requirement:

$$\gamma(x_2) - \gamma(x_1) = \frac{2\pi\phi_A}{\phi_0} \tag{S2}$$

Where ϕ_A is the magnetic flux through a loop connecting x_1, x_2 and extending across the junction length d into the superconductors up to the London penetration depth λ on either side (see Fig. **S3**b). For convenience we denote $L \equiv 2\lambda + d$.

Now we address our specific geometry. For this device we use $NbSe_2$ of thickness around 12-20 layers (≈7-13 nm). Fifteen layers are clearly seen in cross-section TEM measurement (Fig. **S3**c), below 3-5 nm of oxide; also from optical images, $NbSe_2$ thickness may vary slightly throughout the sample (Fig. **S3**e). The junction, of length d=140 nm in the direction of the current flow (y axis), has an MLG weak link of width $W_{MLG} \approx 1.45\mu m$ and an FLG weak link of width $W_{FLG} \approx 0.45\mu m$ and thickness h=2.4 nm (8 layers), their centers separated by a distance $2\delta \approx 2.7\mu m$. We define the x axis in the plane of the MLG/FLG flakes, perpendicular to current flow, and z perpendicular to the MLG/FLG plane. The magnetic field $B_{||}$ we refer to as "in-plane" is oriented parallel to the mean SQUID plane: the plane connecting the center of the MLG and FLG flakes. This plane is at a small angle $\tan(\theta) = \frac{h}{2\delta}$ with respect to the x axis. The field referred to as $B_\perp$ is perpendicular to $B_{||}$. This choice in alignment of $B_{||}$ maintains the peak of the central SQUID oscillation at $B_\perp = 0$ regardless of the applied $B_{||}$ (as described in Section S1). The applied field in x,z coordinates is as follows:

$$B_x = B_{||}\cos(\theta) - B_\perp \sin(\theta) \approx B_{||}, B_z = B_{||}\sin(\theta) + B_\perp \cos(\theta) \approx B_{||}\sin(\theta) + B_\perp \tag{S3}$$

The approximations are to the first order in the small parameters θ and $B_\perp/B_{||}$. The corresponding wave-numbers are:

$$k_1 = \frac{2\pi B_x(2\lambda + d)}{\phi_0}, k_2 = \frac{2\pi B_z(2\lambda + d)}{\phi_0} \tag{S4}$$

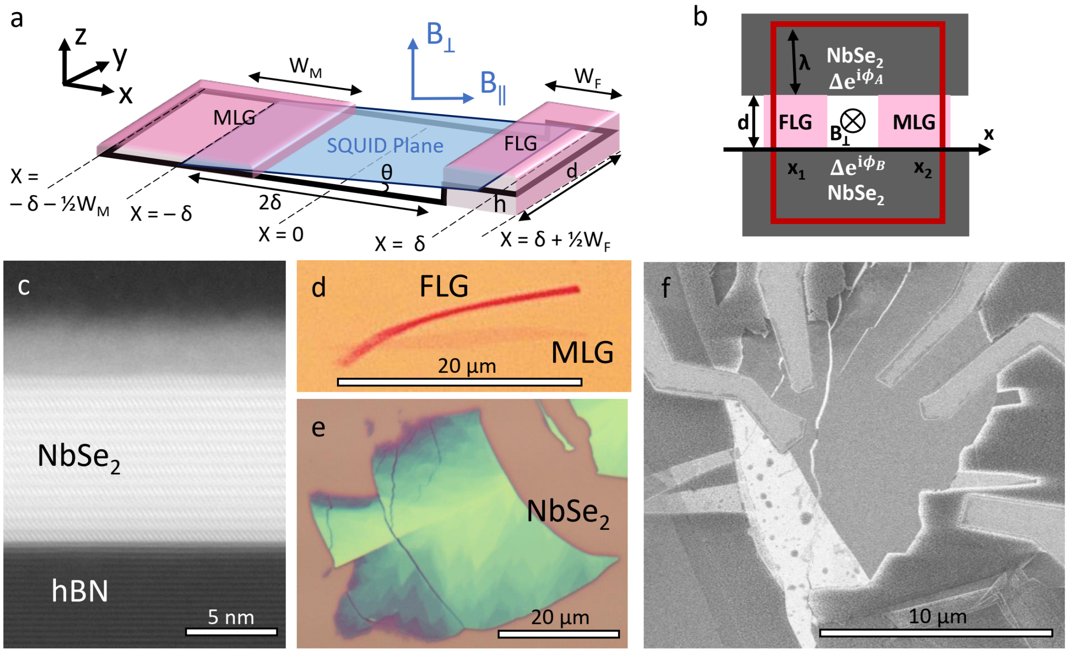

Supplementary Figure S3: **a.** Schematic illustration of FLG and MLG parallel weak links of different thicknesses and widths W_F,W_M respectively. Directions x and y are in the plane of the flakes, z is perpendicular. Mean SQUID plane is shown in blue, at an angle $\tan(\theta) = h/2\delta$. $B_{||}$ is parallel to the SQUID plane and $B_\perp$ is perpendicular to it. **b.** Schematic showing $B_\perp$ flux through one possible current circulation path, with an area $(2\lambda + d)|x_2 - x_1|$. Crack length d is in the direction of current flow. **c.** Cross-section TEM measurement of $NbSe_2$ on hBN. The top 4 nm of $NbSe_2$ are oxidized (amorphous, gray color), underneath are 15 visible layers **d.-e.** Enhanced contrast optical microscope images of graphene and $NbSe_2$ flakes, respectively, exfoliated on SiO_2 **f.** SEM image of the device.

We define a reference phase $\gamma_0 = \gamma(x = 0)$, with $x = 0$ at the center of the junction, such that the centers of the MLG and FLG flake are both at the same distance δ from zero. Finally, the critical current is given by integrating over the current density $J_0(x)sin(\gamma(x))$. We take care that the accumulated phase difference around a closed loop is always 0, accounting for the B_x flux exiting through loops with a vertical portion formed by the step h between the MLG and FlG:

$$I_C(B_\perp) = \max_{\gamma_0}\left(\int_{-\infty}^{\infty} J_M(x)\sin(\gamma_0 + k_2 x) + J_F(x)\sin(\gamma_0 + k_2 x - k_1 h)dx\right) \tag{S5}$$

The integral can be written as the imaginary part of a complex exponential, and the whole expression becomes a Fourier transform:

$$I_C(B_\perp) = \max_{\gamma_0} \mathrm{Im}\left(e^{i\gamma_0}\int_{-\infty}^{\infty}(J_M(x) + J_F(x)e^{-ik_1 h})e^{ik_2 x}dx\right) = \left|\mathcal{F}\big(J_M(x) + J_F(x)e^{-ik_1 h}\big)\right| \tag{S6}$$

To get a simple "zero-order" analytical expression for the critical current in our junction, we normalize the interference pattern by dividing by $I_C(B_\perp = 0)$, and assume a constant current density in each channel, with the ratio $\frac{J_F}{J_M} \equiv f$ an unknown parameter. Normalization of $I_C(B_\perp = 0)$ implies that $J_F W_F + J_M W_M = 1$, and leaves us with the following current densities expressed in terms of f, W_F, W_M:

$$J_F = 1/(W_F + 1/f * W_M); J_M = 1/f * J_F; \tag{S7}$$

Thus we obtain:

$$I_C(B_\perp) = \left| \mathcal{F}\left(J_M \text{rect}\left(\frac{x+\delta}{W_M}\right) + J_F \text{rect}\left(\frac{x-\delta}{W_F}\right) e^{-ik_1 h} \right) \right| \tag{S8}$$

And the analytical expression for the interference pattern:

$$\begin{aligned} \frac{I_C(B_\perp)}{I_C(B_\perp = 0)} &= \sqrt{I_F^2 + I_M^2 + 2 * I_F * I_M * \cos(k_2 * 2\delta + k_1 h)} \\ I_F &\equiv J_F W_F * \text{sinc}(k_2 W_F) \\ I_M &\equiv J_M W_M * \text{sinc}(k_2 W_M) \end{aligned} \tag{S9}$$

The angle of the SQUID plane with respect to the MLG and FLG plane translates into a phase difference between the two channels. Following is a tabulation of the fit parameters $W_M, W_F, 2\delta, \theta, h$ and their errors, extracted from a Matlab non-linear least squares fit of data shown in main text Figs. 3, 4 to the analytical model. The extracted dimensions may be compared to the measured dimensions given above. Note that there are different combinations of slightly different parameter values that can also yield a similar fit, therefore the true fit error is larger than the error bars given in the table.

V_g	$B_\parallel$	W_F	W_M	2δ	θ	h
0 V	0 T	330 ± 10 nm	1260 ± 25 nm	2720 ± 15 nm	X	X
-30 V	0 T	310 ± 20 nm	1590 ± 40 nm	2740 ± 20 nm	X	X
30 V	0 T	300 ± 15 nm	1600 ± 55 nm	2620 ± 30 nm	X	X
0 V	0 T	310 ± 50 nm	1500 ± 60 nm	2600 ± 35 nm	X	X
0 V	2.49 T	210 ± 20 nm	315 ± 40 nm	2500 ± 15 nm	0.024 ±0.0003°	1.1 ± 0.01 nm

Table S1: Extracted parameters from fitting data in main text Fig. 3,4 to the analytical model.

S4 Supplementary Section: Maximum entropy reconstruction of the current profile via Markov Chain Monte Carlo simulated annealing

In order to extract the current distribution in greater detail, we postulate an initial current density profile sampled at N discrete spatial points and subject to physical constraints, and calculate the corresponding interference pattern. We then adjust the density profile sequentially to obtain the best least-squares fit of the calculated interference pattern to the data, subject to a maximum entropy constraint. This is done via Markov chain Monte Carlo simulated annealing.

We begin by guessing a current density vector sampled at N points $\bar{J}_0 = [J_0(x_1), J_0(x_2)...J_0(x_N)]$ normalized such that $\sum_n J_x(x_n) = 1$. Valid guesses are constrained such that non-zero current density

can exist only within the MLG and FLG channels, and current reversal (negative $\bar{J}_0$) is disallowed. We then calculate the corresponding interference pattern based on equation S9, with the perpendicular field sampled at M points $\bar{B}_\perp = [B^1_\perp, B^2_\perp ... B^M_\perp]$, $B_\parallel$ set to some value, step height $h = 1$ nm and junction length $L = 2.2\mu m$ extracted from the analytical fit described in Section S2. Explicitly, we define the matrix element $A^m_n = \exp{(i\frac{2\pi}{\phi_0}LB^m_z x_n)}$ for $x_n < 0$, and $A^m_n = \exp{(i\frac{2\pi}{\phi_0}L(B^m_z x_n - B^m_x h))}$ for $x_n > 0$. We then compute:

$$I^{calc}_C(B^m_\perp) = \left|\sum_n A^m_n J_0(x_n)\right| \tag{S10}$$

In order to quantify the fit of our guess $\bar{J}_0$ we calculate the least squares difference of $I^{calc}_C(B^m_\perp)$ with respect to the measured interference pattern $I^{meas}_C(B^m_\perp)$:

$$\chi^2(\bar{J}_0) = \sum_{m=1}^{M} (I^{calc}_C(B^m_\perp) - I^{meas}_C(B^m_\perp))^2 \tag{S11}$$

We then sequentially adjust the fit by employing a Metropolis algorithm Markov chain Monte Carlo (MCMC) process which samples possible $\bar{J}_0$ configurations and assigns them a free energy reflecting a competition between the goodness of fit χ^2 and the entropy.

$$F(\bar{J}_0) = \chi^2(\bar{J}_0) + \lambda \sum_{n=1}^{N} J_0(x_n) \ln{(J_0(x_n))} \tag{S12}$$

Samples are correspondingly weighted with the standard Boltzmann weight $e^{-\beta F}$. The first term in the free energy penalizes a large deviation of the fit from the measurement, the second (entropy) term penalizes non-uniformity of the postulated current distribution, and the hyper-parameter λ tunes between them. A finite "temperature" $T = \beta^{-1} > 0$ introduces noise to the equilibrium current distribution but allows the algorithm to consider corrections to $\bar{J}_0$ which result in energy loss, and thus helps to avoid converging to local minima of χ^2. In order to find the minimum of F, we employ simulated annealing, increasing the inverse temperature from 0 to β linearly with each Monte Carlo (MC) step. We ensure that $\bar{J}_0$ remains normalized by making changes in discrete units of size ΔJ; a unit removed from site x_i must be added to some other site x_j. The steps of the algorithm are as follows:

1. Make an initial guess $\bar{J}_0$ obeying normalization and constraints.

2. Calculate $F(\bar{J}_0)$

3. Choose x_i, x_j at random from among the sites at which $\bar{J}_0$ is allowed to be non-zero. Propose $J'_0(x_i) = J_0(x_i) + \Delta J, J'_0(x_j) = J_0(x_j) - \Delta J$ so that the total current density is conserved.

4. If either $J'_0(x_i), J'_0(x_j)$ is negative, return to step 3. Otherwise, continue.

5. Calculate the new free energy $F_{new}(\bar{J}'_0)$

6. If $F_{new} < F$, accept new current density and return to step 3 updating $\bar{J}_0 \to \bar{J}'_0$. Otherwise, accept change $\bar{J}_0 \to \bar{J}'_0$ with probability $e^{-\beta_i(F_{new}-F)}$. The temperature at step i is given by $\beta_i = \beta/K * i$, where K is the total number of MC steps.

7. Return to step 3 and iterate K times.

We have freedom to change our initial guess $\bar{J}_0$, and to tune the hyper-parameters $\lambda, \beta, N, \Delta J, K$. The x range of the simulation is defined between $\pm 2\mu m$, while the current in the MLG/FLG channels is bounded within the widths determined from the SEM measurement. Fourier uncertainty indicates that an interference pattern with N nodes yields a spatial resolution of $\frac{W}{N}$; that is, we can choose N evenly spaced discrete points to sample within the overall width W of the current carrying channels. However our fitting method is not an inverse Fourier transform; it introduces additional information through geometrical constraints as well as the maximum entropy constraint. Increasing the number of sampling points helps better fit the width and separation of the channels, while the maximum entropy constraint smooths any sharp spatial features. Thus, we choose $N = 50$, a few times larger than the bandwidth. We use a uniform initial distribution $\bar{J}_0 = \frac{1}{N^*}$, with N^* being the number of sample points allowed to carry current.

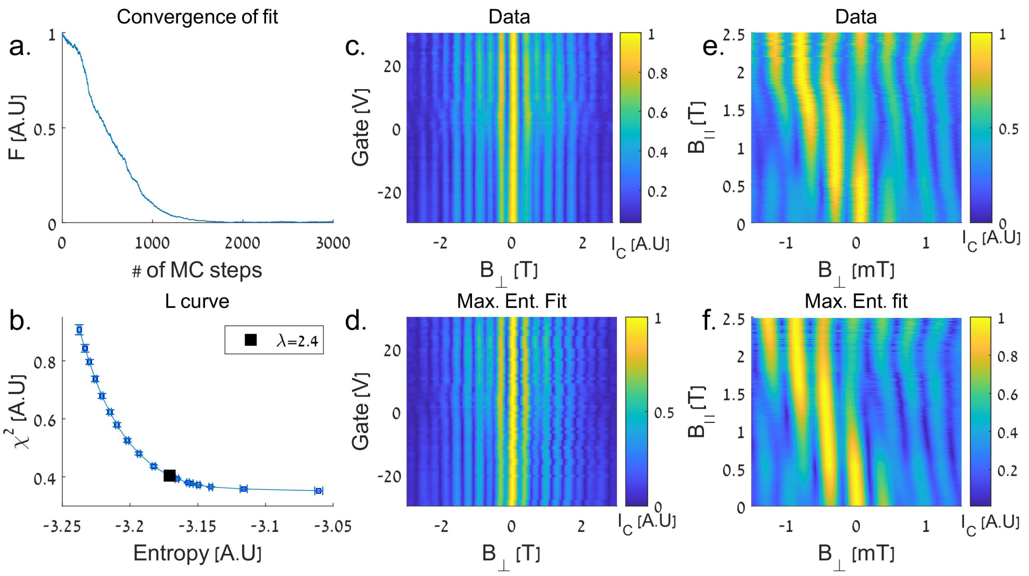

Supplementary Figure S4: **a.** Convergence of the free energy as a function of MC steps **b.** The L curve, a parametric plot of χ^2 and entropy for different values of λ, with chosen $\lambda \approx 2.4$ marked **c.** Normalized I_C (color scale) vs. $B_\perp$ and V_G **d.** Normalized I_C (color scale) vs. $B_\perp$ and gate calculated using Eq. S6, with current density extracted from panel (a) by the Monte Carlo method **e.** Normalized I_C (color scale) vs. $B_\perp$ and $B_{||}$ **f.** Normalized I_C (color scale) vs. $B_\perp$ and $B_{||}$ from eq. S6, with current density extracted from panel (c) by the Monte Carlo method

At zero magnetic field and maximal charge carrier density (V_G=-1V) we have measured a stable, nearly symmetric interference pattern with many lobes (see main text Fig. 3). We use this pattern to tune all of the hyper-parameters of the fitting algorithm. To tune the parameters $\beta, \Delta J$ we set $\lambda = 0$ and try several initial guesses for $\beta, \Delta J$ in powers of 10 before settling on $\beta = 10000, \Delta J = 0.001$ to obtain a convergence of F in a few thousand MC steps (see **S4**a). We note that any $10^3 < \beta$ and $\Delta J < 10^{-2}$ would work as well. We then choose $K = 5000$, several thousand MC steps after convergence. Finally,

we tune λ by studying the L curve (see **S4**b), and choosing $\lambda = 2.4$ which provides a trade-off between decrease in goodness of fit and increase in entropy. Any λ in the vicinity (up to ≈ 6) gives a qualitatively similar current distribution, with smaller λ generating a noisier distribution and larger λ providing a poorer fit. As a sanity check, the spatial features of the current distribution generated by the fit do not have significant features on a length scale finer than that offered by the bandwidth of the original signal, as can be seen in Figs. 3, 4 of the main text. These parameters, chosen once based on the best data set, were then used to fit all of our measured data. See for example measured data of I_C vs. $B_\perp$ and gate voltage (**S4**c) and I_C vs. $B_\perp$,$B_\parallel$) (**S4**e), and compare to the fit in panels d,f. As the MLG current channel grows narrow at high $B_\parallel$ (see main text figure 4), the pre-defined geometric constraint of the width of the MLG flake provides more freedom, leading to reduction in the quality of the maximum entropy fit.

We note that there can in principle be multiple current distributions which give a comparable fit to the interference pattern, due to the loss of phase information. However, fitting three different experimental repetitions of the $B_\parallel$ measurement, changing the fitting hyper-parameters, changing initial conditions, etc. in the above described fitting procedure always results in similar current distributions if we use λ in the vicinity of the optimal λ determined by the L curve.

S5 Supplementary Section: Current density extraction using the Wiener Khinchin theorem

The Wiener Khinchin theorem states that the energy spectral density of a function and its autocorrelation $C(l)$ are Fourier transform pairs. In our case, $|I_C(B_\perp)|^2$ is the energy spectral density of $J_0(x)$, and thus:

$$F(|I_C(B_\perp)|^2) = C(l) = \int_{-\infty}^{\infty} J_0^*(x) J_0(x+l) dx \tag{S13}$$

Consider a two channel device, with the current density in the first channel very sharp and narrow, approximated by the Dirac delta function located at $x = a$, and the current density in the second channel given by some function F of finite width W centered at $x = -a$. The current density is thus: $J_0(x)=\delta(x-a) + F(x+a)$. The autocorrelation of the current density in this case is:

$$C(l) = \int_{-\infty}^{\infty} \delta(x-a)\delta(x-a+l) + F(x+a)F(x+a+l) + F(x+a)\delta(x-a+l) + F(x+a+l)\delta(x-a) dx \tag{S14}$$

The first two terms give some function centered at $l = 0$. If we assume W<a, this function extends no further than $l = \pm W$. The second two terms give $F(2a \pm l)$: this is the current density of the second channel, centered at shifts equal to the distance between the two channels, $l = \pm 2a$ (and mirrored with respect to l around $l = 2a$). In our case the FLG is only a few times narrower than the MLG, and carries a similar current density. In this instance, the autocorrelation convolves the FLG and MLG densities, resulting in a feature which qualitatively resembles the MLG current density "smeared" at the scale of the FLG width, and centered at $l = -2\delta$ equal to the distance between the centers of the two channels.

Note that the calculated auto-correlation functions plotted in figures 3,4 in the main text are smoothed as a result of zero-padding before calculating the discrete Fourier transform of $|I_C(B_\perp)|^2$.
